# Traumatic Fracture in a Patient with Osteopoikilosis

**DOI:** 10.1155/2014/520651

**Published:** 2014-11-16

**Authors:** Adeline Du Mortier, Pierre-Louis Docquier

**Affiliations:** ^1^Cliniques Universitaires Saint-Luc, Service de Chirurgie Orthopédique et Traumatologique, Avenue Hippocrate 10, 1200 Brussels, Belgium; ^2^CARS (Computer Assisted Robotic Surgery), Institut de Recherche Expérimentale et Clinique (IREC), Université Catholique de Louvain, Tour Pasteur +4, Avenue Mounier 53, 1200 Brussels, Belgium

## Abstract

We report a case of traumatic humeral neck fracture occurring in a patient with osteopoikilosis after a motorcycle accident. The radiograph revealed the fracture but also multiple bone lesions. A few years before, the patient had been operated for a maldiagnosed chondrosarcoma of the humeral head. Osteopoikilosis is a rare benign hereditary bone disease, whose mode of inheritance is autosomal dominant. It is usually asymptomatic and discovered incidentally on radiograph that shows the presence of multiple osteoblastic lesions. It can mimic other bone pathologies, in particular osteoblastic metastases. Osteopoikilosis is a diagnosis that should be kept in mind to avoid misdiagnosis, particularly with regard to cancer metastasis. This disorder does not require any treatment and complications are rare. However, there may be associated anomalies that require follow-up.

## 1. Introduction 

Osteopoikilosis (OPK) is a rare bone disease whose prevalence is estimated at 1 per 50,000. It occurs at any age and in both sexes [1–3]. It is a hereditary disease, autosomal dominant, and all affected individuals carry a LEMD3 gene mutation [4–8]. OPK is benign, usually asymptomatic, and is discovered incidentally on radiographs but some cases described in the literature report an association with pain and joint effusions (15–20%), skin manifestations, bone or rheumatic diseases, organ anomalies, and endocrine dysfunctions [4–6, 9]. More extensive radiological investigations make the diagnosis, by showing multiple sclerotic lesions symmetrical with a predilection for epiphyseal and metaphyseal regions of long bones [2, 4, 10]. The differential diagnosis includes other osteoblastic bone diseases, particularly bone metastases. ^99m^Tc bone scan does not show any uptake of radioactive tracer and permits the exclusion of malignancy [4, 6]. This disorder requires no treatment except with regard to the associated manifestations. It must be kept in mind to avoid misdiagnosis (especially that of bone metastases) leading to unnecessary and costly investigations [2, 4].

## 2. Case Presentation

**Figure 1:**
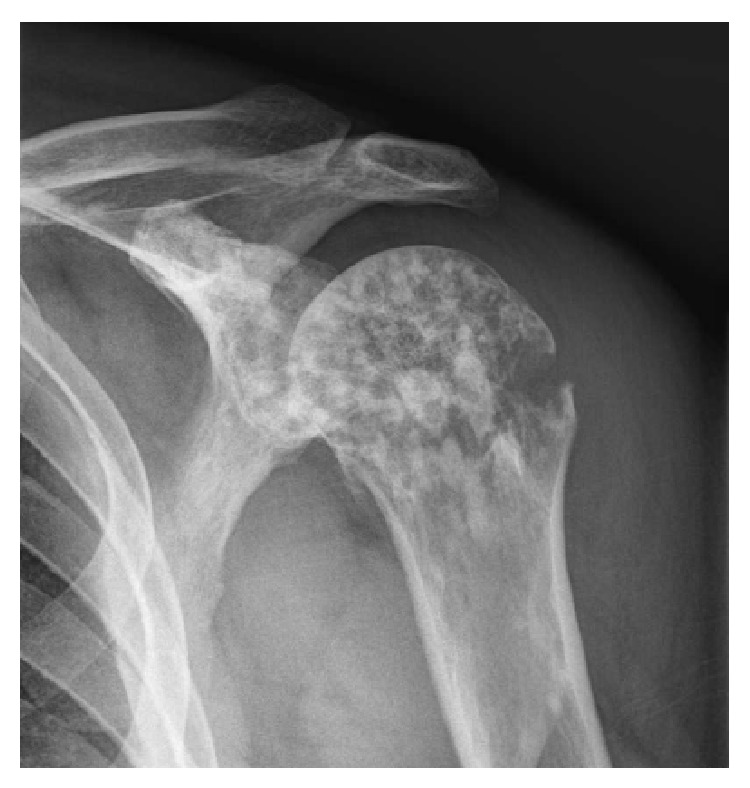
Radiograph of the left shoulder showing a metaphyseal proximal slightly displaced fracture of the humerus and a heterogeneous bone structure with epiphyseal and metaphyseal predominance.

A 37-year-old man presented to the emergency room for pain and functional shoulder impairment after an accidental slip from a motorcycle on the left shoulder, while driving at 40 km/h. On clinical examination, the patient had swelling, spontaneous tenderness, and total functional impairment of the left shoulder. Neurological examination reveals no abnormality. The radiograph of the left shoulder showed a pathological metaphyseal proximal slightly displaced fracture of the humerus and a heterogeneous bone structure with epiphyseal and metaphyseal predominance (Figure 1). At that stage, bone metastases were suspected.

In his history, he had sustained a surgical biopsy for a suspected chondrosarcoma of the right humeral head in another institution at the age of 20 years. Histopathology of the lesion did not reveal the presence of chondrosarcoma. No diagnosis was given to the patient at the time but it was obviously an OPK osteoblastic lesion.

**Figure 2:**
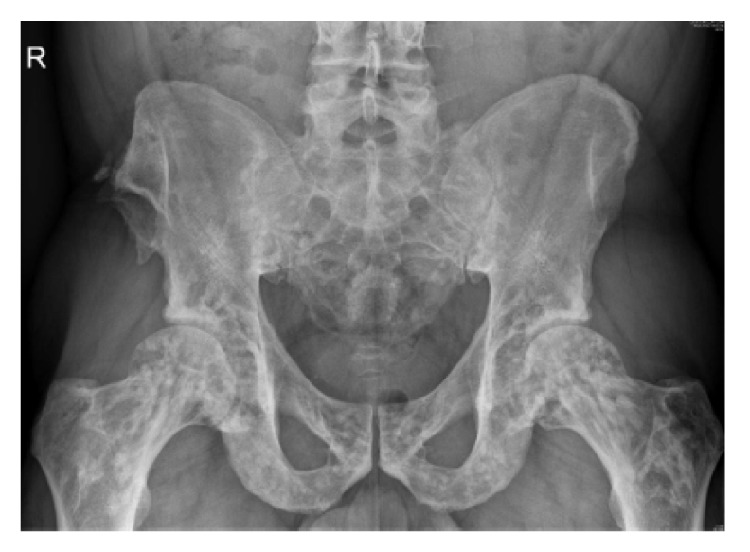
Radiograph of the pelvis showing multiple sclerosis lesions scattered in the pelvis and the upper end of the femur.

**Figure 3:**
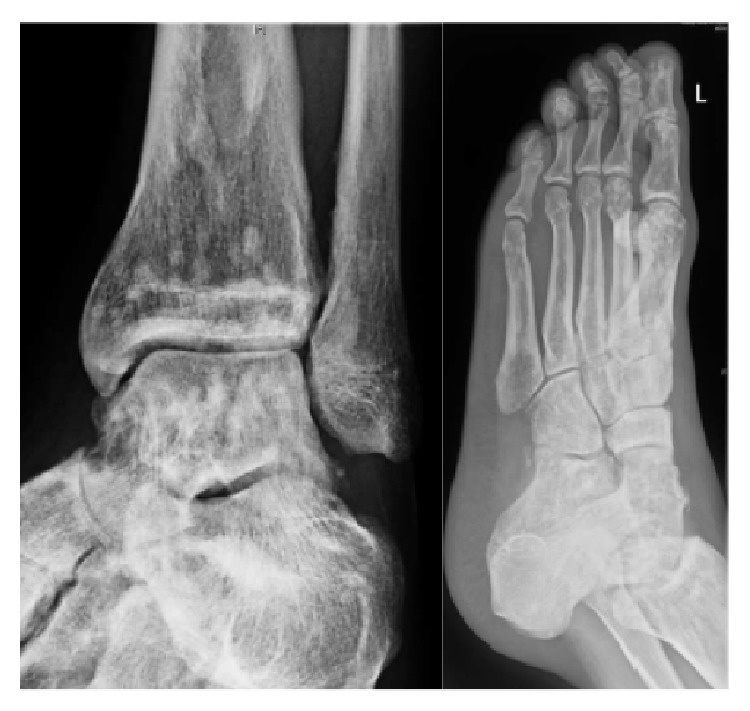
Radiograph of the left ankle and of the left foot showing multiple sclerotic spread lesions, observed at the lower part of the tibia and fibula, of the tarsal bones, metatarsal heads and predominant at the epiphyses of the phalanges.

Thereafter, radiographs of pelvis, right shoulder, right foot, and left foot (Figures 2 and 3) showed multiple sclerotic lesions scattered, small, well-defined, present mainly in epiphyseal and metaphyseal position, evoking an OPK as first hypothesis and making the assumption of secondary osteoblastic metastatic lesions less likely.

**Figure 4:**
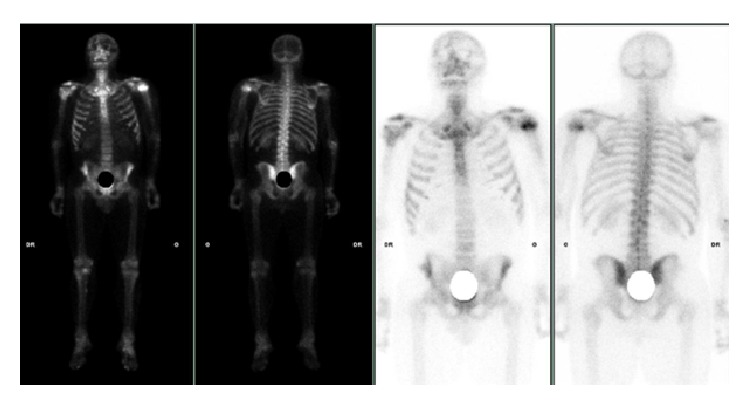
^99m^Tc bone scan revealing uptake only at the site of the fracture of the left humeral head.

Finally a ^99m^Tc bone scan was performed, revealing uptake only at the site of the fracture of the left humeral head, which allowed us to exclude a tumor origin and confirmed the diagnosis of OPK (Figure 4). The treatment focused on the posttraumatic fracture only because regarding OPK, no treatment was required. No delayed union occurred as union was obtained after 6 weeks.

## 3. Discussion

Osteopoikilosis, first described in 1915 by Albers-Schönberg, is rare asymptomatic sclerosing bone dysplasia of benign origin also known under the name of spotted bone disease or osteopathia condensans disseminata and characterized by defective endochondral bone maturation process [2, 3].

This rare disorder, whose prevalence is estimated at 1/50 000, is seen in both men and women and at any age. No studies have formally demonstrated a higher prevalence in either sex. Some suggest that the prevalence is the same in both sexes while others suggest that it is higher with men. It was also suggested that higher frequency among men may result from a referral bias in the literature because men are more likely than women to present to the hospital with traumatic injuries requiring radiologic investigation [1–3].

OPK is typically an asymptomatic condition discovered incidentally on radiologic examination but it can be associated with other abnormalities. In 15–20% of patients mild articular pain and joint effusion without any deformity or dysfunction at the location site have also been reported. OPK has occasionally been reported in association with dermatofibrosis lenticularis disseminata, a predisposition to keloid formation, scleroderma-like lesion, plantar and palmar keratomas, rheumatoid arthritis, lupus erythematosus, ankylosing spondylitis, familial Mediterranean fever, synovial chondromatosis, exostoses, melorheostosis, osteitis condensans, Klippel-Feil syndrome, chondrosarcoma, osteosarcoma, giant cell tumor, dwarfism, dystocia, premyelopathic syndrome due to spinal stenosis, coarctation of aorta, double ureter, dacryocystitis, endocrine dysfunction, and dental and facial abnormalities. Association with connective tissue nevi called dermatofibrosis lenticularis disseminata (DLD) was found in nearly 25% of cases and overlapping of OPK and DLD was defined as Buschke-Ollendorff syndrome [4–6, 9].

In our case, the patient was completely asymptomatic and the discovery of OPK was found incidentally on radiograph of the shoulder.

Studies of familial occurrence indicate an autosomal dominant pattern of genetic transmission associated with heterozygous LEMD3 gene mutations responsible for the disease, resulting in abnormality in endochondral bone maturation process. Sporadic forms are also reported. Recently, whole-genome linkage analysis of carriers of the disease resulted in highlighting of a loss-of-function mutation in gene LEMD3 at position 12q13 which is believed to function in bone morphogenetic protein signaling by interacting with the family of SMAD proteins downstream from TGF-beta to regulate bone formation [4–8]. In our patient, no familial similar cases were found.

Radiologically, the lesions are multiple, small (2–10 mm), well-defined, ovoid, or round, appear as dense radiopaque spots, symmetrically, predominantly periarticular and within the epiphyseal and metaphyseal regions, and are scattered throughout the axial and appendicular skeleton. In clinical and radiologic follow-up of OPK, the lesions remain stable [2–4, 10].

In an epidemiological study of familial osteopoikilosis, Benli et al. found a predominance of these lesions in the phalanges of hand (100%) followed by carpal bones (97.4%), metacarpals (92.3%), foot phalanges (87.2%), metatarsals (84.4%), tarsal bones (84.6%), pelvis (74.4%), femur (74.4%), radius (66.7%), ulna (66.7%), sacrum (58.9%), humerus (28.2%), tibia (20.5%), and fibula (12.8%). The lesions are less common in the skull, ribs, vertebral bodies, and mandible [11]. In our case, humerus, femurs, pelvis, tibias, fibulas, tarsal bones, and foot phalanges were involved. We found multiple small, scattered, predominantly epiphyseal and metaphyseal, and symmetrically distributed sclerotic foci.

Histopathologically, the lesions are formed by dense trabeculae of cancellous bone and form a nidus without communication with the bone marrow [6].

The differential diagnosis includes osteoblastic metastases, mastocytosis, tuberous sclerosis, osteopathia striata, melorheostosis, synovial chondromatosis, Paget's disease, sesamoid ossicles, and Ollier's disease. The major differential diagnosis is osteoblastic metastases. These lesions are characterized by asymmetry, a predilection for axial skeleton involvement, osseous destruction, variation in size, and periosteal reaction. In addition, radionucleotide bone scan can help distinguishing OPK from osteoblastic bone metastases but abnormal bone scan does not exclude OPK. In patients with a known or suspected primary malignancy, radionuclide bone scan has a critical role in distinguishing OPK from osteoblastic bone metastases. Typically, there is no increased uptake of the radioactive tracer visible on bone scan in OPK contrary to bone metastases which characteristically produce numerous “hot spots” of increased activity [2, 4, 6].

The diagnosis is radiological because lesions are very characteristic but ambiguity in this appearance or a history of malignant disease can lead to diagnostic uncertainty and the need for further investigation. It was suggested that the definitive diagnosis of OPK could be made by radiography of both hands, since, in all OPK patients, the metacarpal bones are affected [1, 3, 4].

In our diagnostic approach, we first hypothesized osteoblastic metastases with the first radiograph. We secondly conducted several radiographs that in turn revealed typical lesions of OPK and we excluded a malignancy though bone scan.

Complications are very rare due to the benign nature of OPK. Nevertheless, some complications such as osteosarcoma [12], giant cell tumor [13], and chondrosarcoma [14] have been reported in the literature, but it is not possible to conclude that a causal link exists between OPK and malignant transformation.

OPK does not require any treatment but only follow-up to survey other conditions which may require treatment and the potential risk of malignant transformation [2, 4].

In conclusion, the diagnosis of OPK should be kept in mind to avoid misdiagnosis, especially with regard to bone metastases and to avoid costly and unnecessary investigations. The diagnosis of OPK is radiological because the lesions are very characteristic on X-ray. We should evoke OPK in presence of multiple small radiopaque bone lesions when they are symmetrical, well defined and predominant in the epiphyseal and metaphyseal regions. The radiologist and orthopedic should be aware of this condition to consider this possibility in the differential diagnosis. If it is necessary, a bone scan may be performed to rule out malignancy. OPK is benign and usually asymptomatic and does not require any treatment. Affected individuals live normally. However, conditions associated may require treatment and require follow-up.
